# MYC *cis*-Elements in *PsMPT* Promoter Is Involved in Chilling Response of *Paeonia suffruticosa*

**DOI:** 10.1371/journal.pone.0155780

**Published:** 2016-05-26

**Authors:** Yuxi Zhang, Tingzhao Sun, Shaoqing Liu, Lei Dong, Chunying Liu, Wenwen Song, Jingjing Liu, Shupeng Gai

**Affiliations:** College of Life Sciences, Qingdao Agricultural University, Key Lab of Plant Biotechnology in Universities of Shandong Province, Changcheng Road 700, Qingdao, China; National Taiwan University, TAIWAN

## Abstract

The MPT transports Pi to synthesize ATP. *PsMPT*, a chilling-induced gene, was previously reported to promote energy metabolism during bud dormancy release in tree peony. In this study, the regulatory elements of *PsMPT* promoter involved in chilling response were further analyzed. The *PsMPT* transcript was detected in different tree peony tissues and was highly expressed in the flower organs, including petal, stigma and stamen. An 1174 bp of the *PsMPT* promoter was isolated by TAIL-PCR, and the *PsMPT* promoter::*GUS* transgenic *Arabidopsis* was generated and analyzed. GUS staining and qPCR showed that the promoter was active in mainly the flower stigma and stamen. Moreover, it was found that the promoter activity was enhanced by chilling, NaCl, GA, ACC and NAA, but inhibited by ABA, mannitol and PEG. In transgenic plants harboring 421 bp of the *PsMPT* promoter, the GUS gene expression and the activity were significantly increased by chilling treatment. When the fragment from -421 to -408 containing a MYC *cis*-element was deleted, the chilling response could not be observed. Further mutation analysis confirmed that the MYC element was one of the key motifs responding to chilling in the *PsMPT* promoter. The present study provides useful information for further investigation of the regulatory mechanism of *PsMPT* during the endo-dormancy release.

## Introduction

The mitochondrial phosphate transporter (MPT) shuttles inorganic phosphate (Pi) into the mitochondrial matrix, where Pi is utilized for oxidative phosphorylation to synthesize ATP from ADP. *MPT* encoding genes have been cloned from mammals [1–3], yeast [4], and wood frogs [5] with most studies focusing mainly on the structure and catalytic function of the transporters.

Recently, cloning and characterization of *MPT* were reported in several plants [6–12]. Plant *MPT* genes were identified to be involved in abiotic stress responses, and their expression patterns showed tissue preferences. Birch *Mpt1* was ozone-inducible and highly expressed in the tissue of dividing cells, such as root tips, shoot apices and developing root nodules [6]. *AtMPTs* play an important role in response to salt stress in *Arabidopsis*. Furthermore, with different expression profiles in various tissues and conditions, transcription of *AtMPTs* has been detected in all tissue except siliques [12]. The sequences and structures of 26 potential PT family genes in rice were analyzed, and six *MPT*s also showed tissue preferential expression profiles, among which *OsPT17* and *OsPT19* were differently regulated under hormone treatment conditions. In addition, six putative *cis*-elements were found in all of the *OsPT* genes including ARR1AT, CAATBOX1, CACTFTPPCAL, GATABOX, GT1CONSENSUS and GTGANTG10. Specifically, GATABOX and GT1CONSENSUS are light-responsive *cis*-elements, and CACTFTPPCAL is necessary for carbon metabolism [11]. Current knowledge of *MPT* regulation and the molecular mechanisms mediating its biological functions in plants is still incomplete.

Tree peony (*Paeonia suffruticosa* Andrews) is one of the most well-known horticultural and medicinal plants in the world. One of the main production mechanisms in the tree peony industry, especially for the Spring Festival flower market in China, is forcing culture. Dormancy is a major obstacle for the forced culture of tree peony in winter, and sufficient chilling is an efficient way to break dormancy. Therefore, it is important to determine how chilling induces dormancy release in tree peony. *PsMPT* was previously isolated from the tree peony subtractive cDNA library of burst buds and strongly induced by chilling treatment to promote ATP production during the release of bud dormancy. In addition, ectopic-expression of *PsMPT* in *Arabidopsis* showed that *PsMPT* enhanced ATP synthesis and affected plant growth and development [10]. These results suggested that *PsMPT* plays an important role in energy production during bud dormancy release in tree peony [13]. However, the expression characteristics of *PsMPT* and its regulatory mechanisms are unclear.

In this study, we isolated the promoter of *PsMPT* and constructed *PsMPT* promoter::*GUS* engineered *Arabidopsis*. We investigated: 1) the temporal and spatial characteristics of the *PsMPT* promoter in *Arabidopsis* and *PsMPT* expression in tree peony; 2) how plant hormones and abiotic stresses, including chilling, affects the activity of the *PsMPT* promoter; 3) which one of *cis*-elements among the *PsMPT* promoter is involved in the chilling response.

## Materials and Methods

### Plant materials

Four-year-old tree peonies (*Paeonia suffruticosa* ‘Luhehong’) were obtained from the Tree Peony Research Center of Heze (Shandong, China). According to the method of Huang et al.[10], plants were treated in cold conditions (0–4°C) for 21 days to break bud dormancy, as the daily mean temperature was under 10°C in Qingdao, Shandong, China. The plants were then transferred to a greenhouse (18–22°C, 8-h-light/16-h-dark cycle) to resume growth. Tissues (root, stem, leaf, calyx, petal, stamen and carpel at the early stage of flowering) were collected and stored at -80°C until use. One hundred μmol·L^-1^ ABA and 50 μmol·L^-1^ GA_3_ were applied to non-chilling buds with double-distilled water as the control, and buds were collected after 0, 1, 6, 12, 24 and 48 h. Three replicates (3 plants/replicate) were performed for all treatments.

### Isolation of the *PsMPT* promoter

Genomic DNA was extracted from tree peony buds using the cetyltrimethylammonium bromide (CTAB) extraction method as previously described [14]. DNA samples were qualified photometrically, then checked on agarose gel, and stored at -20°C for use. Based on the cDNA sequence of *PsMPT* (Genbank accession No.: EU072922), three gene-specific primers, SP1, SP2 and SP3, in nested positions were designed with primer premier 5.0 and synthesized (Table 1). The *PsMPT* promoter was amplified using gene-specific primers and four short arbitrary primers (AP) with the Genome Walking Kit (TaKaRa). The PCR products were ligated into the pMD18-T vector (TaKaRa), and sequenced at BGI-Beijing, China.

**Table 1:** The primers used in this paper.

Primers	Sequences (5’-3’)	purpose
SP1	CTGATGTTAGGGTTCTACTTTCCTCTTTCTCTC’	Promoter isolation
SP2	ATGTCTGCGTTACCCAAGGTCGTCCC	Promoter isolation
SP3	ATAGGGCATTCCCAGAAACGATTGTCC	Promoter isolation
FP1	GGGAAGCTTGGGACCCAGTGTGT (*Hin*d III)	Promoter analysis
FP2	GGGAAGCTTTGGGGACTCAATTGT (*Hin*d III)	Promoter analysis
FP3	GCGAAGCTTGATACAATGGGAGAGGAG (*Hin*d III)	Promoter analysis
FP4	GGGAAGCTTGGTCGCATTCGTCG (*Hin*d III)	Promoter analysis
FP5	GGGAAGCTTAGAACAAGAATCGTGGAG (*Hin*d III)	Promoter analysis
FP6	GGGAAGCTTAGCTCGGCATTCAGTG (*Hin*d III)	Promoter analysis
RP	CGAGGATCCCATATCTGATGTTAGG (*Bam*H I)	Promoter analysis
FDP	GGGAAGCTTCATATTATGTCAAATTGG (*Hin*d III)	Motif identification
RM1	TCCAATTTGACATAATATGTTACAAGGT	Motif identification
FM1	TTATGTCAAATTGGAGACTTGATT	Motif identification
GUSFW	AGTGGCAGTGAAGGGCGAACAGT	qPCR of *GUS*
GUSRV	TCAGCGTAAGGGTAATGCGAGGT	qPCR of *GUS*
ActinFW	GAGAGATTCCGTTGCCCTGA	qPCR of *β-actin*
ActinRV	CTCAGGAGGAGCAACCACC	qPCR of *β-actin*
MPTFW	GCTGGAGGAATAATGAGTTGTGG	qPCR of *PsMPT*
MPTRV	GCACCTTGAGCACTGTAACCA	qPCR of *PsMPT*

### Bioinformatics analysis of the promoter sequence

Regulatory elements in the promoter were analyzed using the online program PLACE (http://www.dna.affrc.go.jp/PLACE/) [15, 16] and Plantcare (http://bioinformatics.psb.ugent.be/webtools/plantcare/html) [17].

### Construction of the promoter-reporter plasmids

To construct the binary vector consisting of the β-glucuronidase (GUS) coding sequence driven by the *PsMPT* promoter, a fragment from -1117 to -1 relative to the translation initiation codon was obtained using the high fidelity DNA polymerase and promoter specific primers (FP6 and RP, Table 1). The serial deletions of the *PsMPT* promoter (-909, -621, -574, -421, and -282 to -1) were amplified by PCR with the corresponding forward (FP1, FP2, FP3, FP4, FP5) and reverse primers (RP) that contained a *Hin*d III and *Bam*H I site at the 5' end of each primer, respectively. For chilling response element identification, the MYC element -413 relative to the translation initiation site was subsequently deleted from the P2 construct with the FDP primer, and mutated to form the MP construct by recombination PCR with the FM1 and RM1 primers (Table 1). PCR products were retrieved and cloned into a pMD18-T simple vector (TaKaRa) followed by sequencing conformation at BGI Beijing, China. The *Hin*d III/*Bam*H I digested DNA fragments were inserted into the corresponding sites of pBI121, in place of the deleted CaMV35S promoter upstream of the *GUS* coding sequence. The pBI121 vector with the *CaMV35S*::*GUS* was used as the positive control. The resulting constructs were named P1 (-282), P2 (-421), P3 (-574), P4 (-621), P5 (-909), P6 (-1117), DP (-404), and MP (-574), respectively.

### *Arabidopsis* transformation

*Arabidopsis thaliana* wild-type (Col-0) plants were transformed using the *Agrobacterium tumefaciens* strain EHA105 carrying the above constructs according to the floral dip method [18]. The transformants were screened on MS medium containing 50 mg L^-1^ kanamycin, and positive plants were identified by PCR amplification using GUS specific primers (GUSFW and GUSRV, Table 1). The corresponding T_1_ transgenic seedlings that segregated at a ratio of 3:1 (resistant: sensitive) were selected to propagate T_2_ individuals for further analysis. Five to ten transgenic lines were obtained for every construct.

### Hormone and abiotic stress treatments

The transformed *Arabidopsis* plants grew at 18°C in a controlled growth chamber (16-h-light/8-h-dark cycle), and 5-week-old *Arabidopsis* plants were used to analyze the response of the *PsMPT* promoter to hormone and abiotic stresses. For chilling treatment, plants were cultured under 4°C for 24 h, and those at 18°C were used as controls. Moreover, the inflorescence of 5-week-old *Arabidopsis* plants were sprayed by GA_3_ (50 μmol L^-1^), NAA (100 μmol L^-1^), ABA (100 μmol L^-1^), ACC (250 mmol L^-1^), NaCl (200 mmol L^-1^), mannitol (40 μmol L^-1^) and PEG (100 mmol L^-1^) treatments for 24 h, respectively. Double-distilled water was used as a control. Samples were collected after 0, 1, 3, 6, 12 and 24 h treatments and stored at -80°C.

### GUS histochemical assay and quantitative analysis of GUS activity

GUS activity was determined in tissue extracts using a standard protocol [19]. GUS fluorescence was measured with a Microplate Spectrofluorometer, the data were obtained by subtracting the background 4-methyiumbelliferyl glucuronide of the *PPsMPT*::*GUS* transgenic plants. The average GUS activity was obtained from at least five independent transformants, and each assay was repeated three times. GUS histochemical staining was performed using identified homozygous transgenic plants by a modified Jefferson’s method [19]. In brief, plant tissues were incubated in a 100 mmol L^-1^ sodium phosphate buffer (pH 7.0) containing 0.1% Triton X-100, 10 mmol L^-1^ EDTA, 1 mmol L^-1^ X-gluc and 0.5 mmol L^-1^ potassium ferricyanide at 37°C overnight. The stained tissues were then washed several times with 70% ethanol to bleach the chlorophyll.

### Quantitative real-time PCR (qPCR)

Total RNA was extracted from the inflorescence of *Arabidopsis* and buds of tree peony as previously described [20], and then treated with DNase I (TaKaRa) according to the manufacturer’s instructions. First strand cDNA was synthesized from 2 μg of total RNA using the PrimerScript^™^ RT reagent Kit (TaKaRa). PCR reactions were performed in a 25 μL system including 12.5 μL 2×SYBR Green Master mix (TaKaRa), 300 nmol L^-1^ each primer (Table 1) and 2 μL 10-fold diluted cDNA template. PCR reactions were run in a Roche LightCycler^®^ 480 (Roche, Germany) using the following program: 95°C for 2 min and 45 cycles of 95°C for 5 s, 57°C for 30 s and 72°C for 30 s. The reactions were run in triplicate. The expression was normalized to beta-actin. Quantification of the relative gene expressions was performed using the 2^-ΔΔCt^ method [21]. Statistical analyses were performed using SPSS 13.0 (SPSS, USA).

## Results

### Expression characteristics of *PsMPT* in tree peony

**Fig 1:**
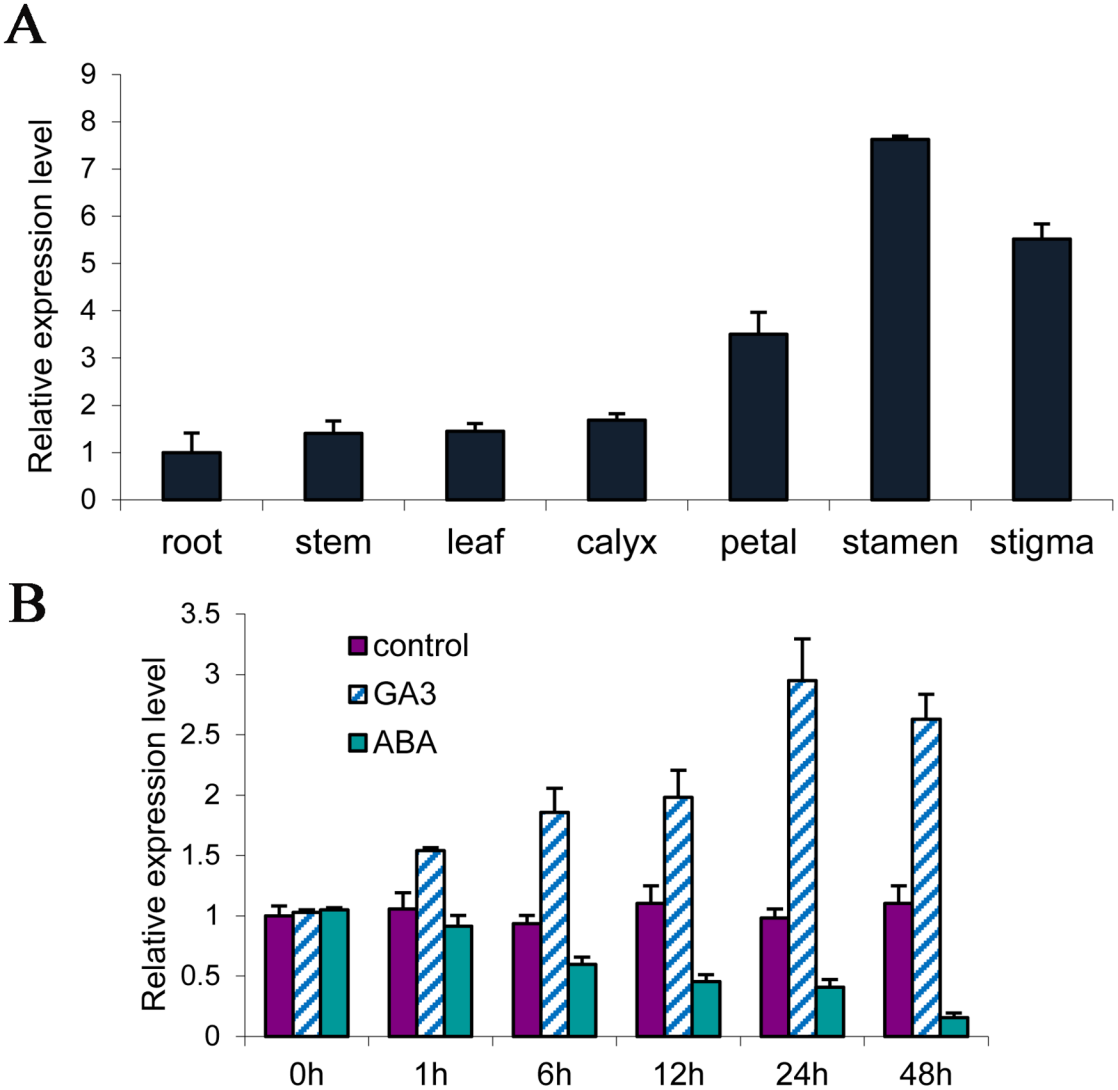
Tissue-specific expression of *PsMPT* in germinated buds (A) and transcriptional levels of *PsMPT* in response of GA_3_ and ABA of tree peony by qPCR (B). 100 μmol L^-1^ ABA and 50 μmol L^-1^ GA_3_ was sprayed to the dormant buds in green house (18–22°C, 8-h-light/16-h-dark cycle). Values are means ±SD of three replicates.

The expression of *PsMPT* was previously reported during dormancy release in tree peony [13]. In this study, the temporal and spatial expression of *PsMPT* was further detected at the early stage of flowering in tree peony. The results of qPCR indicated that the transcription of *PsMPT* was detected in all tree peony tissues; however, the *PsMPT* transcript was very low in root, stem, leaf and calyx, but high in flower organs, including petal, stamen and stigma (Fig 1A). The *PsMPT* transcripts in the stamen were expressed 6-fold as compared to that of the root. The results indicated that *PsMPT* was expressed preferentially in flower organs of tree peony.

The response of *PsMPT* in dormant buds to Gibberellic Acid (GA) and Abscisic Acid (ABA) were analyzed by qPCR. When GA was applied, the *PsMPT* transcript was quickly promoted and peaked at 24 h, then declined slightly. Conversely, ABA application dramatically decreased the expression of *PsMPT* (Fig 1B).

### Isolation of the *PsMPT* promoter and the putative *cis*-acting element

**Fig 2:**
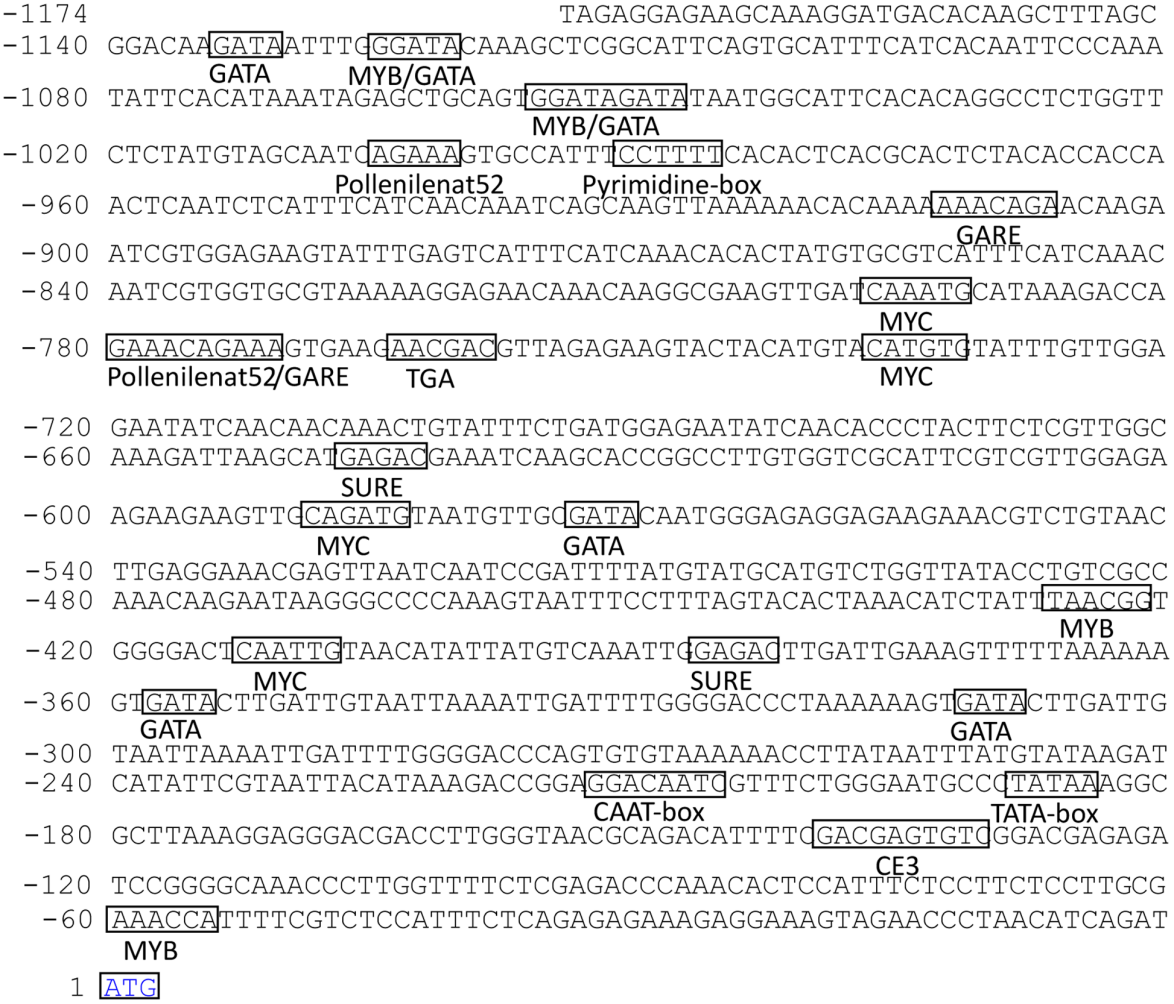
*PsMPT* promoter sequence of the 5’ region upstream from the start codon (ATG) and putative *cis*-elements predicted in the promoter region. Numbers indicate the positions relative to the translation start codon starting from the adenosine (+1). The putative **TATA** box at position -189, CAAT box at position -210, and the ATG start codon is framed in box and denoted with blue color. The other important putative *cis*-elements are framed and labeled below.

In this study, the 1174 bp upstream genomic DNA sequence of *PsMPT* was isolated by TAIL-PCR (Fig 2). The adenosine of the translation initiation codon (ATG) of the *PsMPT* gene was defined as +1 (Fig 2). A motif search was carried out using PLACE and PlantCare to analyze the putative *cis*-elements. As shown in Fig 2, the putative TATA box was found at position -189/-185 and the CAAT box at position -210/-207. In addition, a number of regulatory motifs potentially related to environmental signals were found, which included auxin-, GA- and dehydration-responsive and tissue-specific elements. Among the *cis*-elements, one putative pyrimidine box and two GA-responsive elements (GAREs) were located at -991/-986, -913/-907 and -780/-771, respectively, which were related to GA response and sugar repression [22, 23]. Four putative Myelocytomatosis viral oncogene homolog (MYC) (5’-CANNTG -3’) motifs and four Myeloblastosis viral oncogene homolog (MYB) (5’-WAACCA -3’, 5’-YAACKG -3’ or 5’-GGATA-3’) motifs were located at positions -797/-792, -737/-732, -589/-584, -412/-408 and -1127/-1122, -1056/-1051, -427/-422, -40/-35, respectively. These motifs had previously been identified in response to dehydration in *Arabidopsis* [24–26]. Several MYB and MYC motifs were thought to respond to chilling or freezing in *Arabidopsis* [24–26]. There were two putative sulfur-responsive element (SURE) motifs at positions -647/-643 and -387/-382, and one putative TGA element (auxin responsive element) at position -764/-759. SURE contains the auxin response factor binding sequence [27]. Therefore, *PsMPT* might be regulated by chilling and auxin. Interestingly, eight putative GATA boxes were present in the sequence, which were thought to be involved in tissue-specific expression and light response [28, 29]. Moreover, three putative pollen1lelat52 elements, related to the pollen specific expression [2, 30], were found at -775/-771, -782/-777, and -1005/-1001, respectively.

### Temporal and spatial expression of the *PsMPT* promoter in *Arabidopsis*

**Fig 3:**
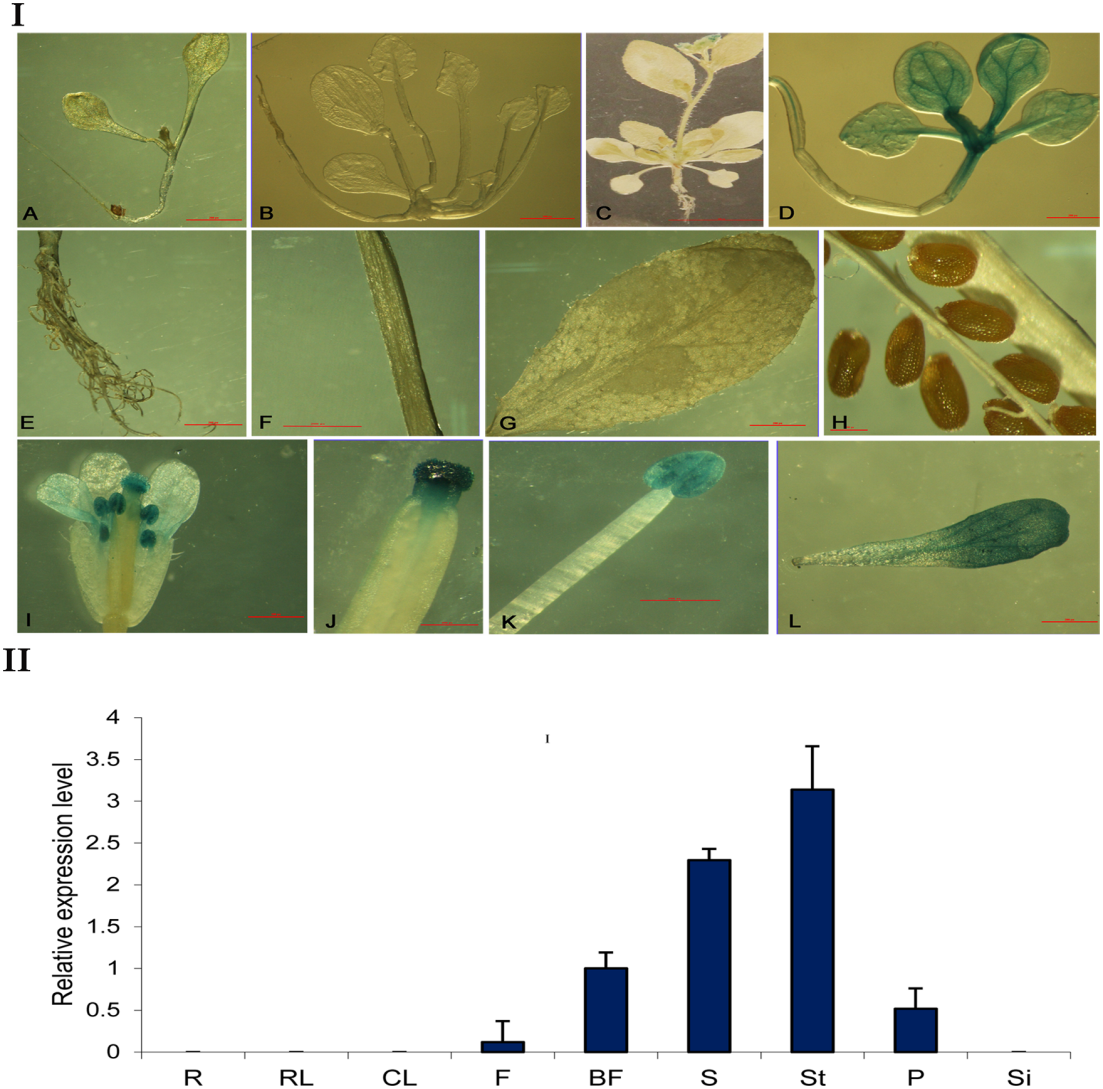
Histochemical localization (I) and tissue-specific expression of *GUS* (II) in transgenic *Arabidopsis thaliana* carrying the *PsMPT* promoter::*GUS* construct. (I)Histochemical localization of GUS expression by staining with X-gluc in transgenic *Arabidopsis thaliana* carrying the *PsMPT* promoter::*GUS* construct. Arrow bar shows GUS staining in flower. **A** 7-day-plants from seeding; **B** 21-day-plants from seeding; **C** 28-day-plants from seeding; **D** Positive control (Ca35S promoter driven); **E** roots; **F**: stem; **G** leaf; **H**: mature silique; **I** flower; **J** stigma; **K** stamen; **L** petal. (II)Total RNA was isolated from roots (R), rosette leaf (RL), cauline leaf (CL), flower bud (F), bloomed flower (BF), stamen (S), stigma (St), petal (P) and siliques (Si) of 35-day-old transgenic plants from seeding. The transcriptional levels were analyzed by qPCR using GUS gene-specific PCR primers, which were normalized with beta-actin. Values are means ± SD of three replicates.

To identify the expression patterns of the *PsMPT* promoter, the promoter::*GUS* chimeric construct (*PPsMPT*::*GUS*) was transformed into *Arabidopsis*, and histochemical GUS staining was carried out in various organs throughout plant development (Fig 3I). These results showed that GUS activity was not detected in the seedling tissues (Fig 3IA and 3IB) but were observed in the flower organs (Fig 3IC, 3IE–3IG). GUS activity in transgenic plants was more pronounced in the stigma and stamen compared with the sepals (Fig 3II–3IL). GUS staining was not observed in the silique and seeds (Fig 3IH). These results suggested that *PsMPT* is preferentially expressed in the flower tissues.

qPCR was performed to evaluate the spatial expression of the *PsMPT* promoter. The results showed that the *GUS* transcript was only detected in the flower (Fig 3II). No transcript of *GUS* was detected in the roots, rosette leaves, cauline leaves and silique. *GUS* transcript levels in bloomed flowers were approximately 10-fold higher compared with flower buds. In bloomed flowers, the most abundant expression was found in stigma, followed by in stamen, with the lowest amount in the petals. In summary, the GUS staining results are in accordance with that of qPCR. The GUS reporter did not appear in the immature flower buds possibly due to the low abundance of the transcripts. The flower-specific expression characteristics of the promoter implied that *PsMPT* might participate in plant anthesis and gametophyte development.

### Responses of the *PsMPT* promoter to hormones and abiotic stresses

**Fig 4:**
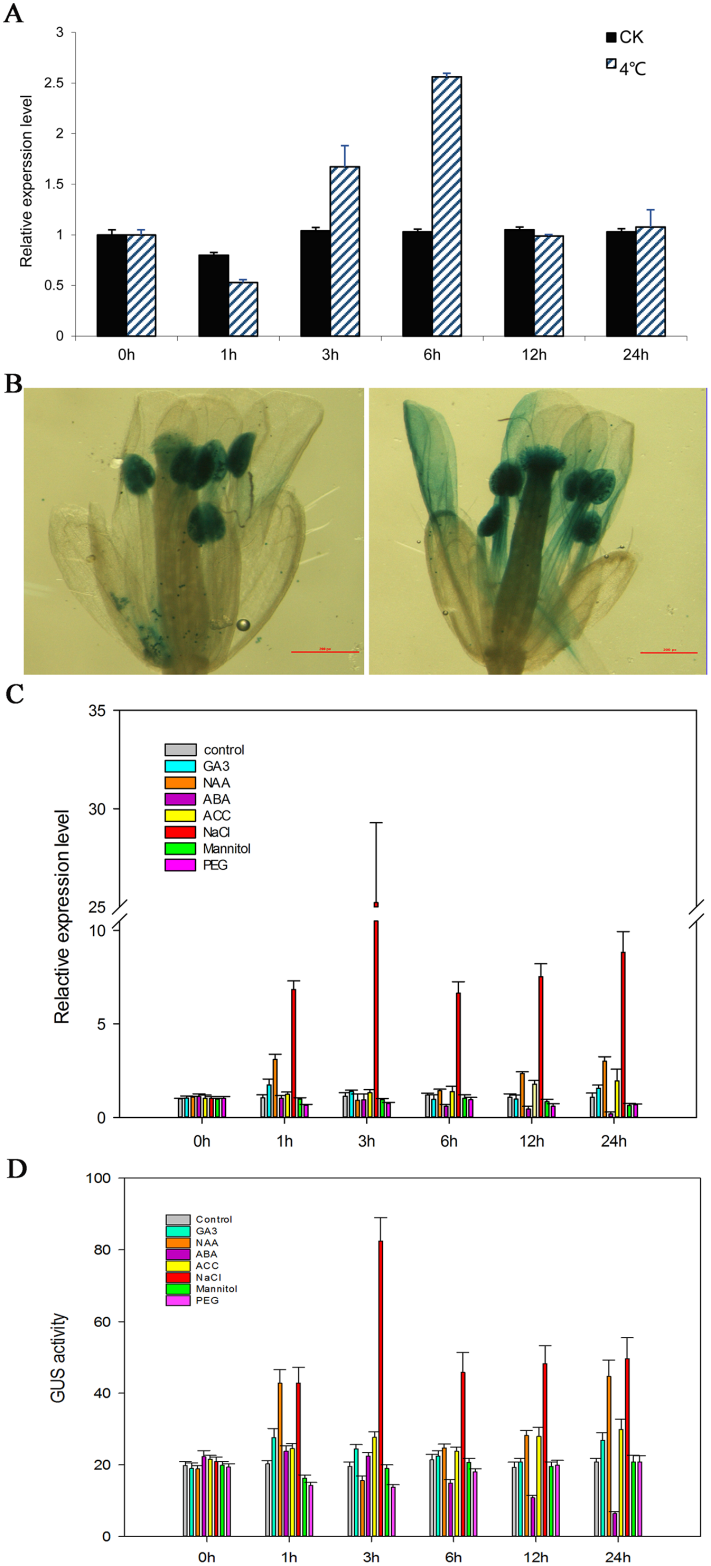
Relative expression levels of *PsMPT* promoter in response to hormone and various abiotic stresses treatments in transgenic Arabidopsis plants. (A) *GUS* expressions when exposed to 4°C temperature. Plants were transferred to a cold chamber maintained at 4°C, and the control grew at 18°C. Error bars represent ±SD. (B) GUS staining of transgenic *Arabidopsis* grew at 18°C (left) and at 4°C for 6 h (right). (C) *GUS* expressions were measured by qPCR using 35-day-old plants from seeding. 50 μmol L^-1^ GA_3_, 100 μmol L^-1^ NAA, 100 μmol L^-1^ ABA, 250 mmol L^-1^ ACC, 200 mmol L^-1^ NaCl, 40 μmol L^-1^ mannitol and 100 mmol L^-1^ PEG was sprayed to the inflorescence at 18°C, and double-distilled water treatment was used as control. (D) *GUS* fluorescence (nmol L^-1^ min^-1^ μg protein^-1^) were measured by a Microplate Spectrofluorometer using 35-day-old plants from seeding. 50 μmol L^-1^ GA_3_, 100 μmol L^-1^ NAA, 100 μmol L^-1^ ABA, 250 mmol L^-1^ ACC, 200 mmol L^-1^ NaCl, 40 μmol L^-1^ mannitol and 100 mmol L^-1^ PEG was sprayed to the inflorescence at 18°C, and double-distilled water treatment was used as control.

The transgenic plants carrying the *PPsMPT*::*GUS* cassette were treated with hormones and abiotic stresses, and the transcription of *GUS* was evaluated by qPCR, respectively. Overall, *GUS* activity changed rapidly and fluctuated during the entire period for all of the treatments (Fig 4). Chilling increased the *GUS* transcript during 3 h to 6 h after treatment, with a peak approximately 2.5-fold higher at 6 h (Fig 4A), which was also verified by GUS staining (Fig 4B). The results of qPCR showed that GA_3_ and NAA treatments enhanced *GUS* expression at 1 h, then decreased, followed by another peak at 24 h. ACC accelerated the transcript of *GUS* during the entire process. Conversely, ABA inhibited *GUS* expression throughout the process. In addition, the *GUS* transcript was continuously decreased by mannitol and PEG until 3 h, followed by a slight increase at 12 h and 24 h; however, the *GUS* transcript levels were lower than that of control. Notably, NaCl dramatically enhanced GUS activity, and it reached a peak at 3 h with an approximate 25-fold increase (Fig 4C). The results of GUS activity were consistent with *GUS* expressions of qPCR (Fig 4D).

### Identification of chilling response elements

**Fig 5:**
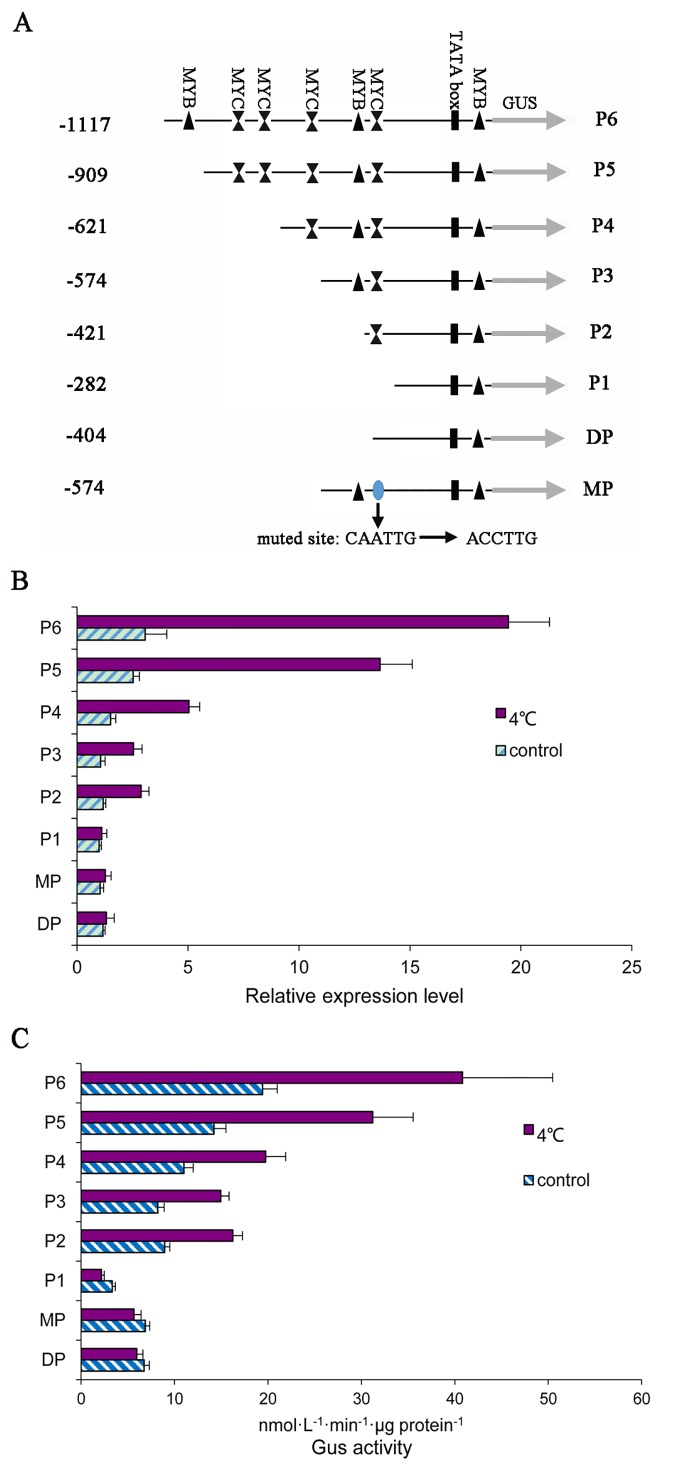
Assays for GUS expression driven by the series *PsMPT* promoter. (A) Schematic diagram of the *PsMPT* promoter deletions and mutation that were used to analyze the activity of different fragments of the *PsMPT* promoter. All fragmented promoter were fused to a GUS reporter gene. (B) Quantitative analyses of GUS expression in transgenic plants driven by deletion or muted constructs of *PsMPT* promoter in response to chilling. (C) GUS activity in transgenic Arabidopsis plants. The inflorescence of 5-week-old Arabidopsis plants was used as material, and five independent lines for every treatment.

As reported previously, *PsMPT* was chilling inducible. Furthermore, *PsMPT* was involved in chilling induced dormancy release in tree peony. To confirm the *PsMPT* promoter region involved in the chilling response, a number of truncated promoter fragments (P1 (-282), P2 (-421), P3 (-574), P4 (-621), P5 (-909) and P6 (-1117)) were isolated and fused to the *GUS* reporter gene into pBI121 vector (Fig 5A). Transgenic Arabidopsis plants with each promoter-GUS construct were generated. The transcription levels of GUS were detected by qPCR (Fig 5B). In the transgenic plants, the highest level of *GUS* expression was detected in the engineered Arabidopsis with the P6 construct, which contained the full-length *PsMPT* promoter (−1117/−1). GUS transcript decreased in order from the P6 to P1 construct, and P2 and P3 had similar abundance. These results suggested that the transcription enhancer might exist in the upstream of the *PsMPT* promoter.

We also detected *GUS* expression of the successive deletions at 4°C for 6 h compared to 18°C (Fig 5B). After exposure to 4°C, the *GUS* transcripts were up-regulated in the P2 to P6 constructs. The largest increase in *GUS* abundance was observed in plants with the P6 construct (6.34-fold). The next-largest increase was observed in the P5 construct (-909/-1, 5.40-fold). Similar enhancement was observed in the P2 and P3 constructs with 2.45 and 2.41-fold, respectively. However, the P1 construct (-282/-1), which only contained basic transcriptional elements, could not induce GUS expression at 4°C. These results indicated that the −421/−282 region of the *PsMPT* promoter could lead to efficient chilling induction. Bioinformatics analyses showed that a MYC *cis*-element existed in this region, and several MYCs were found within the promoter. We speculated thus that the MYC elements in the promoter might be involved in the chilling response.

To verify our hypothesis, another deletion construct (DP) (-404) and one mutated construct (MP) (-574) were constructed (Fig 5A). In the DP construct, the MYC element located at -412 in the promoter was deleted. When exposed to 4°C, no significant difference was observed as compared to that of 18°C treatment, which indicated that there was a loss of function for chilling induction with the MYC element deleted in the *PsMPT* promoter (Fig 5B). In the MP construct where the MYC element (-412) was mutated from CAATTG to ACCTTG, a complete elimination of the chilling response was observed (Fig 5B).

To confirm the results obtained by qPCR, a quantitative measurement of GUS activity was performed using the series constructs, P1-P6, DP and MP. Consistent with the results obtained by qPCR, the GUS activity was observed to increase from the P2 to P6 after exposed to 4°C, and no difference was observed in the P1, DP and MP. In summary, we concluded that the MYC element involved in chilling treatment responses (Fig 5C).

## Discussion

MPT can shuttle inorganic phosphate (Pi) into the mitochondrial matrix, where ATP is synthesized. Thus, MPT plays a key role in cellular ATP regeneration. ATP is essential for almost all biological processes in the cell, and *MPTs* have been reported to be involved in abiotic responses [6, 12], bud dormancy release, growth and development [13]. Although several *MPT*s have been cloned and functionally annotated, their characteristics and regulatory mechanisms are poorly understood.

We previously cloned *PsMPT*, a chilling induced gene, in tree peony, which accelerated ATP synthesis and dormancy release [13]. In this study, we isolated the *PsMPT* promoter and detected its activity using GUS as the reporter in transgenic *Arabidopsis*. GUS-staining and qPCR of the transgenic plants revealed that the *PsMPT* promoter was preferentially expressed in the flower, mainly in stamen and stigma. Therefore, the tissue-specific expression may be related to the putative GATA boxes and pollen1lelat52 elements founded in the promoter [30, 31]. The results suggested that *PsMPT* might play an important role during gametophyte development, pollination and fertilization, and Pi might be transported to reproductive organs during the reproductive development stage. Differing from ectopic expression of the *PsMPT* promoter, *PsMPT* mRNA was detected in all tissues of tree peony by qPCR. This discrepancy has also been reported in mice [32, 33] and is believed to be related to different biological species or incomplete isolation of promoter sequences.

The temporal and spatial expression of *MPT* has been reported in several plants, and all of the results showed tissue-specific expression. Birch *MPT1* was highly expressed in tissues containing dividing cells [6]. There were six *MPT* members in rice, and a microarray analysis also revealed tissue-specific expression [11]. In *Arabidopsis*, the transcription of the *AtMPT1* was pronounced in the stamens of flowers, and *AtMPT2* mRNA was abundant in rosette leaves; whereas, *AtMPT3* was strongly expressed in leaves and weakly expressed in the roots and flowers [12]. Overall, the spatial pattern of *PsMPT* was similar to *AtMPT1*. In contrast, *PsMPT* showed only 51% sequence identity with *AtMPT1*, and 79% sequence similarity with *AtMPT3* (S1 Table). The organization of promoters between *PsMPT* and *AtMPT*s was also compared, and large differences were observed (S1 Fig). Similarly discrepant MPT sequences and expression patterns between tree peony and Arabidopsis were also found in grape and *Arabidopsis* [11]. Taylor et al. investigated the relationship between the promoter and coding sequence selective constraint and suggested that they were generally uncorrelated [34], which implied partially independent evolution of promoters and their coding sequences between species. Therefore, we speculated that the discrepancy might be due to species-specific independent evolution of MPTs and their promoters.

The response of the *PsMPT* promoter to abiotic stress and hormones was analyzed by qPCR. *GUS* transcript increased during chilling treatment in the transgenic plants driven by the *PsMPT* promoter. In addition, the *PsMPT* promoter was also induced by salt, GA_3_, ACC and NAA, while ABA, mannitol and PEG suppressed its activity. In rice, *OsPT19* was suppressed by five hormone treatments including ABA, 2, 4-D, GA_3_, KT, and NAA, whereas *OsPT17* was induced by the hormone treatments with NAA or GA_3_ [11]. These responses might be due to the *cis*-elements in the promoter, for instance, the putative pyrimidine box and two GARE motifs to GA response, putative TGA and SURE elements to NAA response, and MYB and MYC to abiotic stress response, such as chilling. Interestingly, NaCl treatment could significantly increase the expression of the reporter gene with a maximum of approximately 25-fold. Similar results were reported for *Arabidopsis* [12].

Among all of the factors influencing promoter activity, chilling and GA_3_ treatments are of interest because they effectively accelerate the dormancy release in winter [35–38]. Huang et al. found that *PsMPT* was induced by chilling [13]. Interestingly, it was reported that the chilling-induced expression of *PsMPT* was not maintained after being transferred to a greenhouse (18–22°C) when less than 21 days of chilling were applied. On the other hand, the levels of *PsMPT* transcripts remained high with a 21 d or longer chilling duration after returning to growth temperature [13]. In this study, ectopic expression analyses provided more evidence that the *PsMPT* promoter could be induced by chilling. We speculated that the early increase of the *PsMPT* transcript might be induced by chilling, and GA production might be a downstream effect of chilling, as proposed for dormant seeds [39, 40]. Meanwhile, chilling temperature was reported to enhance the accumulation of endogenous GA, and exogenous GA could partially replace chilling to accelerate endo-dormancy release [41]. In this study, we found that exogenous GA could activate the expression of the *PsMPT* promoter. Therefore, the reactivation of the *PsMPT* transcripts might be due to the high endogenous GA induced by sufficient chilling when transferred to growth condition. Buds chilled for less than 21 days had relatively low GA levels that could not activate the *PsMPT* expression required for the recovery of plant growth ability.

Considering the central role of *PsMPT* in energy metabolism during dormancy release, it is important to elucidate how chilling accelerates *PsMPT* expression. It is well-known that the transcription of mRNA is mainly regulated through the cooperation of transcript factors and corresponding *cis*-elements. Several *cis*-elements have been identified to be involved in chilling or cold responses, such as ABRE (ABA responsive element), DRE/CRT (dehydration-responsive element/C-repeat element, A/GCCGAC), MYB, MYC, and the E-box [24, 26, 42, 43, 44, 45]. Bioinformatics analysis of the isolated *PsMPT* promoter showed that four MYB and four MYC elements were present upstream of the promoter, which might be responsible for the chilling response. Based on the location of the MYC and MYB elements, deletion experiments were conducted to identify the candidate chilling response elements in the promoter. *GUS* expression and activity revealed that the P2 construct containing one MYC element (-412/-408, CAATTG) effectively responds to chilling, and the addition of a MYB element (P3 construct) did not improve the chilling response ability. When MYC was deleted or mutated, the chilling response character abated. Alternatively, increase of MYC elements in the P4, P5 and P6 constructs enhanced the chilling response activity, indicating there was an additive effect of MYC elements in the chilling response. This study demonstrates that the MYC elements in the *PsMPT* promoter play a crucial role in the chilling responses.

In conclusion, we isolated the *PsMPT* promoter in tree peony and found that it is a floral-preferential promoter. The promoter of *PsMPT* responded to chilling, ACC, PEG, NaCl, mannitol, auxin and GA. Deletion and mutation analyses demonstrated that the MYC *cis*-element functioned in the chilling response. This work provides useful information for further investigation of the regulatory mechanisms of *PsMPT* promoter during endo-dormancy release.

## Supporting Information

S1 Fig Organization of the PsMPT and AtMPTs promoters. (DOCX)

S1 Table The identity (%) between PsMPT protein and the *Arabidopsis* and rice MPTs. (DOC)
