# Supplementary material for: MYC cis-Elements in PsMPT Promoter Is Involved in Chilling Response of Paeonia suffruticosa
Source: PLoS One. 2016 May 26;11(5):e0155780. doi: 10.1371/journal.pone.0155780 (PMC4882030; doi:10.1371/journal.pone.0155780)
Supplement: S1 Fig — (DOCX) [file pone.0155780.s001.docx]

**S1 Fig. Organization of the promoters of PsMPT and AtMPTs.**
Promoters of 1174 bp, 737 bp, 688 bp, 1026 bp were analyzed by Plantcare and PLACE as described in the materials and methods. *Cis*-elements marked with graphics were listed below. For simplification, some motifs were combined, such as light responsive, MeJA responsive and so on. MYB, MYC, light responsive (not shown in the promoter of PsMPT), GA-response elements were all presented in the three promoters.
