# Supplementary material for: MYC cis-Elements in PsMPT Promoter Is Involved in Chilling Response of Paeonia suffruticosa
Source: PLoS One. 2016 May 26;11(5):e0155780. doi: 10.1371/journal.pone.0155780 (PMC4882030; doi:10.1371/journal.pone.0155780)
Supplement: S1 Table — (DOC) [file pone.0155780.s002.doc]

**S1 Table The identity (%) between PsMPT protein and the *Arabidopsis* and rice MPTs.**

|  | AtMPT1 | AtMPT2 | AtMPT3 | OsMPT1 | OsMPT2 | OsMPT3 | OsMPT4 | OsMPT5 | OsMPT6 |
| --- | --- | --- | --- | --- | --- | --- | --- | --- | --- |
| PsMPT | 51% | 69% | 79% | 81% | 78% | 79% | 71% | 72% | 55% |
